# Mr. Atkinson's Medical Bibliography

**Published:** 1834-04-01

**Authors:** 


III.—(BIS.)

MEDICAL BIBLIOGRAPHY: A and B. By *James Atkinson*, Senior Surgeon to the York County Hospital and the York Dispensary, &c. &c. Royal 8vo, pp. 382. London, Churchill, 1834.

DURING the whole of *our* bibliographical labours, which have not been few, we have never encountered so singular and remarkable a book as that now before us. It unites the German research of a Plouquet, with the ravings of Rabelais—the humour of Sterne with the satire of Democritus—the learning of Burton, with the wit of Pindar,—we mean, of course, PETER Pindar! We regret, almost as much as the author, that there is no probability of a completion of the work. As a large volume is occupied with two letters of the alphabet, and as the compiler can be little short of the age allotted to man, we can hardly expect that even a second volume shall ever be edited by Mr. Atkinson! This melancholy prospect has not escaped our author. In a note at page 49, the following passage occurs, and will bear us out in our sombre forebodings.

"But I must here, however, request to make a digression. I must indeed; and in favour of this immortal Haller, since time and tide will stay for no man. Incerta omnibus spes est vitæ, senibus non incerta solum sed omnino vix ulla superest.—Haller. Nor little can I expect to live so long, as to enable me in possibility, to proceed in my alphabet to letter H. And is not vita brevis, and tempus arctum? Are not libri multi, and nummi parci? And have I not well surveyed the mouth of that molaris fellow, old edax rerum? He is to me, reader, of frightful aspect. For teeth beset his mouth and palate in all directions—the most cruel and diversified. He is carnivorous, nay, omnivorous; with most formidable crotophite muscles; which befit him to destroy, not only poor me, but any thing." 49.

The work is addressed to "all idle medical students in Great Britain," and is embellished with a humorous figure of the OS SACRUM! The wit of this may not be detected at first sight; it may thus be explained—

To
All Idle Medical Students
in
Great Britain,
Sit
SACRUM.

Although the author has drawn inexhaustible stores of learning from our German literati, he seems to have an insuperable antipathy to the German language—and indeed to all *dead* tongues.

"I have studiously avoided much communication with the Germans, or with their most excellent authors, and, in most humble deference, with the dead languages ; for, every thing dead, except victuals, I abhor. And, notwithstanding, I possess Noëhden's German Grammar or Syntax; yet, I know not so much of that tongue as of a neat's tongue: or, as a child just born. Nay, in point of guttural expression, I know less. The German tongue is to me most odontolgoid and difficult. And although I am very sensible that mere dint of application may convert an alphabet into a language, yet all the patent presses in a printing-office, could not squeeze one syllable of the German accent out of my tongue."—*Preface*, i.

The plan of the laborious compilation under review is to give a catalogue of each author's writings, and also all the editions of them in chronological order, with a few brief remarks interspersed among the editions. This task must have occupied the author many years, and a drier or more monotonous avocation could scarcely be imagined! Thus the mere catalogue of Aristotle's writings and the different editions, fills eight royal octavo pages—and this small portion of the book alone might seem to require half a life-time to ferret out every printer and commentator, from Joann Mentelius, of Mantua, in 1470, down to Camus, of Paris, in 1783!

It would appear that the dullness of the pursuit had the effect of accumulating the vivacity of the compiler, which every now and then bursts from its place of confinement and surprizes the reader by the contrast of quaint humour with dusty catalogues, monotonous dates, and barbarous names. Thus, speaking of a very fine edition of Aristotle, in Gothic characters, with figured capitals, long lines, catch-words, and registrum cartharum, printed in 1496, in Venice, and preserved in the York Minster library, our facetious bibliographer observes:—" Some philosophical snail, however, has left vestiges in writing of his slow commentating track upon the margin of the first folium of Lib. i. de Cœlo. He would have been a long time, if we may judge by this progress, in creeping up to this ceiling, or cœlo."

Passing over several ancient physicians, we have the following remarks on Asclepiades and Avicenna.

"Asclepiades was, by report, a wild, erratic, vagabond son of physic, but a talented man. Fragments of his works and genius are recorded by the above authors. He derided and lived without physic,—I mean, without taking it—to the age of 80.—Wonderful!

In some respects I doubt, Avicenna had chosen him for a pattern. He prescribed wine for himself, and for his patients—sometimes to excess. Pliny relates, that he died from a fall.

Of him says the comical poet:

'Wherefore to cure all his bruises and knocks,
He was used to drink vinum orthodox;
And one day did it so effectually,
He dislocated his epistrophe.' "* 14.

Speaking of the *Tetrabiblos* of Ætius, as an excerpt from the writings of the ancients, our humorous author has a sly hit at some modern bibles.

"Now there are too many tea-tray bibles in our days; which makes them worth nothing. Every old washerwoman has her bit of butter sent to her, from

* Epistrophe is the swivel of the neck.

the huckster's shop, wrapt up in one of the leaves. And I have occasionally seen them, like so many muscæ volitantes in the turbid humours of a diseased eye, scudding about in all directions, or swimming down the channel of a common sewer." 44.

Although the *tea-tray* pun is not the brightest piece of wit in the world, yet it is as good as many of those witticisms which amuse our Sunday readers of hebdomadal newspapers.

The writings of Albertus Bolstalius furnishes our author with several passages of sarcastic commentaries—and some severe squibs on the Bishops of old. These, however, we shall pass over, as the Bishops have enemies enough in our days.

When speaking of John de Gaddesden's "Rosa Anglica," he quotes a modest prefix of honest John, which runs thus:—

> "Et sicut Rosa excellit omnes flores;
> Ita iste liber excellit omnes practicas medicinæ."

Among the various proofs of the antiquity of the venereal disease, the following is no bad specimen:—

"John de Gaddesden, about the year 1340, writes of infection thus, 'Primo notandum quod ille qui timet de excoriatione et arsura virgæ post coitum statim lavet virgam cum aqua mixta aceto, val cum urina propria, et nihil mali habebit' —indeed!

And also de ulcere Virgæ, 'Sed si quis vult membrum ab omni corruptione servare cum a muliere recedit quam forte habet *suspectam* de immunditie, (is this from uncleanliness or infection?) lavet illud cum aqua frigida mixta cum aceto, vel urina propria intra vel extra præputium.' How simple the cure by ablution —for a Jew!" 97.

Our author, though a Roman Catholic, does not appear to be very strait-laced in religious creeds. The following passage will make most of our readers smile.

"But in religious matters I must affect nothing, for I am no Unitarian, no Biarian, no Trinitarian, no in unum Congregarian, no methodist or Ranter, no Protestant: except that I protest merely to be, a (bon) Roman Catholic, as the best Catholicon going. For sic itur (I am told) ad astra—and who travels safer? Nay I will not even condescend to be a free thinker, though I doubt a free writer. My own free wit I fear, (like too much common salt) when in full dose will make you sick; but when I wish to give any for a cure; to be administered pure; I steal, or borrow, or run a-tic. Since, however, every thing now is 'No Popery,' to what a miserable existence are we poor Papists doomed! Hard is the fate of him, whose preservation and every other ration seems to depend upon his chylification. Whose class even as an animal can scarcely be identified. He is obliged, from the temperate laws of his religion, to be continually varying his dietetic circumstances. He is not strictly speaking, a carnivorous animal, although he be man, and as man should be: because he is often interdicted eating meat. He is not a high-bred, but a hybrid Christian. If he be allowed 'permissu superiorum,' to eat this meat, (rarely venison), once a week, the next, he dares scarcely chew the cud, or if none be there to chew, he downright starves. Now, there might be some prospect of a blissful year for him, were there luckily a leap year of Lent, or rather a leap over lent year; but no —the vermin papist is like the horse in a mill, or like the maggot in a deaf nut, who works incessantly, and in vain, round the dark concave of a melancholy pabuless circle, neither with beginning nor end—sad emblem of Eternity!" 123.

We must draw to a close our brief notice of this humorous and unique performance, by only one more extract. After alluding to a quack doctor of the name of M'Adam, who appears to have been "Yorkshire too," but with whom Mr. Atkinson says he never exchanged cards, he introduces the following anecdote of—

"MC'ADAM,
The Doctor Viarum, or Road Doctor.

This appellation is not synonymous with *make* Adam, as we shall see below; for, Adam primus had no father.

The present Mc'Adam is a hardened character, and must not be forgotten. He is famous at this day for making hard, and mending soft, turnpike roads.

He could not have learnt this art from his ancestors, as they resided in a garden, and never went but once (post haste) out of it! Before that period they walked on turf, or fortunately, had gone on velvet, until Satan was the ruin of them. But he may have acquired it from the Romans, whose military roads were much on this construction.

ANECDOTE.

Being one of the commissioners for a turnpike road, near York, we were letting the toll and repairing of the road, to the best bidder. Each candidate brought some pretensions of skill in the art of road making; one of them, (a rough subject) was asked, if he was acquainted with the new mode of Mc'Adam? Mc'Adam! why gentlemen, he replied, I made roads before Adam was born!" 171.

We must now take leave of our facetious bibliographer, strongly recommending his book to the attention of all our melancholy or hypochondriacal brethren—for if it does not make their sides shake with laughter, they are too far gone in the "blue devils," to leave any hope of recovery.

---

